# Pioglitazone Improves Mitochondrial Organization and Bioenergetics in Down Syndrome Cells

**DOI:** 10.3389/fgene.2019.00606

**Published:** 2019-06-28

**Authors:** Nunzia Mollo, Maria Nitti, Lucrezia Zerillo, Deriggio Faicchia, Teresa Micillo, Rossella Accarino, Agnese Secondo, Tiziana Petrozziello, Gaetano Calì, Rita Cicatiello, Ferdinando Bonfiglio, Viviana Sarnataro, Rita Genesio, Antonella Izzo, Paolo Pinton, Giuseppe Matarese, Simona Paladino, Anna Conti, Lucio Nitsch

**Affiliations:** ^1^Department of Molecular Medicine and Medical Biotechnology, University of Naples Federico II, Naples, Italy; ^2^Department of Morphology, Surgery and Experimental Medicine, University of Ferrara, Ferrara, Italy; ^3^Institute of Experimental Endocrinology and Oncology, National Research Council, Naples, Italy; ^4^Department of Biology, University of Naples Federico II, Naples, Italy; ^5^Department of Neuroscience, Reproductive and Odontostomatological Sciences, University of Naples Federico II, Naples, Italy; ^6^Department of Biomedicine, University of Basel, Basel, Switzerland

**Keywords:** Down syndrome/therapy, pioglitazone, energy metabolism, oxidative stress, mitochondrial dysfunction, mitochondrial dynamics

## Abstract

Mitochondrial dysfunction plays a primary role in neurodevelopmental anomalies and neurodegeneration of Down syndrome (DS) subjects. For this reason, targeting mitochondrial key genes, such as *PGC-1α/PPARGC1A*, is emerging as a good therapeutic approach to attenuate cognitive disability in DS. After demonstrating the efficacy of the biguanide metformin (a *PGC-1α* activator) in a cell model of DS, we extended the study to other molecules that regulate the *PGC-1α* pathway acting on *PPAR* genes. We, therefore, treated trisomic fetal fibroblasts with different doses of pioglitazone (PGZ) and evaluated the effects on mitochondrial dynamics and function. Treatment with PGZ significantly increased mRNA and protein levels of PGC-1α. Mitochondrial network was fully restored by PGZ administration affecting the fission-fusion mitochondrial machinery. Specifically, optic atrophy 1 (*OPA1*) and mitofusin 1 (*MFN1*) were upregulated while dynamin-related protein 1 (*DRP1*) was downregulated. These effects, together with a significant increase of basal ATP content and oxygen consumption rate, and a significant decrease of reactive oxygen species (ROS) production, provide strong evidence of an overall improvement of mitochondria bioenergetics in trisomic cells. In conclusion, we demonstrate that PGZ is able to improve mitochondrial phenotype even at low concentrations (0.5 μM). We also speculate that a combination of drugs that target mitochondrial function might be advantageous, offering potentially higher efficacy and lower individual drug dosage.

## Introduction

Over the last years, several drugs and nutraceuticals have been tested, mostly in the Ts65Dn mouse model of Down syndrome (DS), aimed at rescuing or attenuating deficits in learning and memory. More than 20 molecules have been successfully identified to restore hippocampal deficits in adult mice (Gardiner, 2015). The results of these preclinical trials suggest that the amelioration or prevention of cognitive deficits in people with DS may be possible, thus paving the way to clinical trials many of which are still in progress. Leitmotiv of these investigations was that improving cognitive function would have enormous beneficial consequences for individuals with DS, increasing their potential to participate in society more fully and independently.

In human DS cells we and others have documented an impairment of mitochondrial function with decreased mitochondrial redox activity and decreased levels of mitochondrial electron transport enzymes, altered mitochondrial morphology and increased fragmentation (Piccoli et al., 2013).

Several studies demonstrated that mitochondrial dysfunction plays an important primary role in both altered neurodevelopment and neurodegeneration (Khacho and Slack, 2018). This evidence strongly supports the hypothesis that a mitochondria targeted therapy might attenuate cognitive disability in DS.

Among the molecular mechanisms responsible for mitochondrial dysfunction in DS, we found that the dosage-related over-expression of a corepressor mapping to the chromosome 21 (Hsa21), namely *NRIP1*, inhibits the activity of the transcriptional activator *PGC-1*α (Izzo et al., 2014), a gene that orchestrates mitochondrial biogenesis and function (Scarpulla et al., 2012). Indeed, *PGC-1*α activity is decreased in cells and tissues of DS individuals (Conti et al., 2007; Piccoli et al., 2013; Izzo et al., 2014), as well as in brain tissues from Alzheimer’s disease (AD) patients (Petrozzi et al., 2007), suggesting that similar molecular pathways are involved in DS and AD neurodegeneration (Coskun and Busciglio, 2012).

PGC-1α is a modulator of mitochondrial phenotype, its activity might be targeted through either PGC-1α activators, successfully tested in mouse models for other diseases (Lagouge et al., 2006; Dong et al., 2007), or agonists of the peroxisome proliferator-activated receptors (PPARs), which have demonstrated to attenuate mitochondrial dysfunction in AD mouse models (Nicolakakis et al., 2008; Johri et al., 2012).

We have recently evaluated a PGC-1α activator, the biguanide metformin, in a DS cell model (Izzo et al., 2017b). Metformin, commonly used in clinical practice in type 2 diabetes as well as in polycystic ovary syndrome and insulin resistance, activates PGC-1α *via* AMPK and SIRT1 post-translational modifications. We demonstrated that metformin induces both the expression and the activity of PGC-1α in human trisomic fibroblasts, promoting mitochondrial biogenesis (Izzo et al., 2017b).

Even though metformin is a very promising molecule, the use of different drugs acting on same targets through different pathways could be eligible as alternative or synergistic approach. For this reason, in this study, we considered drugs that may regulate mitochondrial function by activating *PGC-1α* pathway through the activation of PPARs. Three PPARs have been identified (α/γ/δ), which are ligand-modulated nuclear receptors that regulate gene expression programs of metabolic pathways (Michalik et al., 2006). PPAR-γ has proven to be a promising target for treatment of central nervous system diseases. Agonists of this receptor showed to be effective in ameliorating disease-related symptoms in animal models (Corona and Duchen, 2015). Both *PGC-1α* activators and PPAR-γ agonists regulate the expression of several target genes involved in neuronal survival and neuroprotection by inhibiting mitochondrial dysfunction, oxidative stress, proteosomal dysfunction, autophagy, neuroinflammation and apoptosis (Chaturvedi and Beal, 2008; Landreth et al., 2008; Corona and Duchen, 2016).

Among the PPAR agonists, pioglitazone (PGZ) (ATC code A10BG03), belonging to thiazolidinediones (TZD) (ATC code A10BG), selectively stimulates PPAR-γ (Landreth et al., 2008). PGZ increases the phosphorylation of AMPK and, as consequence, the expression of *PGC-1α* and of multiple genes involved in mitochondrial function (Coletta et al., 2009). This drug attenuates mitochondrial dysfunction in animal models of central nervous system injury improving mitochondrial ATP production and oxygen consumption (Nicolakakis et al., 2008; Escribano et al., 2009; Sauerbeck et al., 2011), prevents the loss of dopaminergic neurons (Breidert et al., 2002) and reduces iNOS induction and oxidative stress in mouse models of Parkinson’s disease (PD) (Hunter et al., 2008). PGZ is known to normalize hyperglycemia-induced intracellular ROS and mtROS production (Fujisawa et al., 2009). It restores brain ATP levels (Garcia-Bueno et al., 2007) and increases mitochondrial DNA content, oxygen consumption rate (OCR), PGC-1α and TFAM levels in human adipose tissue and in the neuronal-NT2 cell line (Bogacka et al., 2005; Ghosh et al., 2007). In order to test if PGZ is able to counteract mitochondrial dysfunction in DS, we treated trisomic fetal fibroblasts (DS-HFFs) with different doses of PGZ to evaluate the effects on mitochondrial function and morphology.

## Materials and Methods

### Ethics Statement

Four cultures of human fetal fibroblasts, from fetuses with trisomy of Hsa21 at 18–22 gestational weeks, were obtained from the “Telethon Bank of Fetal Biological Samples” at the University of Naples. All experimental protocols were approved by the local Institutional Ethics Committee.

### Samples

Fibroblasts were cultured in T25 flasks (BD Falcon) with Chang medium B + C (Irvine Scientific) supplemented with 1% penicillin/streptomycin (Gibco) at 37 °C in a 5% CO_2_ atmosphere. All analyses described throughout this study were carried out at cell culture passages 4–5.

Analysis of karyotype was performed according to standard methods (Genesio et al., 2011).

### Pioglitazone Treatment

PGZ (Cayman Chemical) was dissolved in dimethyl sulfoxide (DMSO) to a stock solution of 5 mM and added to the cell growth medium at the final indicated concentrations. Fresh PGZ was added every 24 h for 72 h. In untreated control cells an equal volume of DMSO was added every 24 h.

### RNA Extraction and Quantitative RT-PCR

Total RNA from each sample was extracted using TRIzol reagent (Gibco/BRL Life Technologies, Inc., Gaithersburg, MD) and was reverse-transcribed using the iScript cDNA Synthesis kit (Bio-Rad Laboratories, Inc., Hercules, CA, USA). Quantitative real time polymerase chain reaction (qRT-PCR) was performed using SsoAdvanced universal SYBR Green supermix on a Bio-Rad iCycler CFX96 Touch Real-Time PCR Detection System according to the manufacturer’s protocols. Primer pairs (MWG Biotech, Ebersberg, Germany) were designed using the Primer 3 software (http://bioinfo.ut.ee/primer3-0.4.0/primer3; last accessed date 2015) to obtain amplicons ranging from 100 to 150 base pairs. Primer efficiency was tested generating standard curves for each gene. QRT-PCR results are presented as relative mRNA levels normalized against reference control values. The *GAPDH* housekeeping gene was chosen as reference gene.

### Western Blotting

Western blotting was performed as previously described (Izzo et al., 2017b). Protein extracts, separated by sodium dodecyl sulphate - polyacrylamide gel electrophoresis (SDS-PAGE) and transferred onto Nitrocellulose membranes, were incubated with the following specific primary antibodies: anti-CASPASE 3 (Cell Signaling Technology), anti-PGC-1α (Calbiochem), anti-GAPDH (Cell Signaling Technology), anti-Actin (Sigma), anti-MFN1 (Santa Cruz Biotechnology), anti-MFN2 (Santa Cruz Biotechnology), anti-OPA1 (Santa Cruz Biotechnology), and anti-DRP1 (Cell Signaling). Primary antibodies were detected with the appropriate HRP-conjugated secondary antibodies (Santa Cruz Biotechnology or GE-Healthcare) and revealed by chemiluminescence (Pierce) using digital imaging on a Bio-Rad ChemiDoc XRS apparatus or Fuji X-ray film.

### Analysis of Mitochondrial Network Architecture

Cells, seeded at a density of 50,000 per well onto 25-mm glass coverslips, were allowed to grow for 24 h and then infected with mitochondria-targeted green fluorescent protein (GFP) inserted into an adenoviral vector (Ad-mtGFP Ex/Em: 495/515) as previously described (Izzo et al., 2017b). Protein expression was then allowed for 72 h in the presence or absence of PGZ.

The efficiency of infection was comparable in treated and untreated trisomic cells both in terms of percentage of GFP positive cells (about 80%) and of intensity of fluorescent GFP signal.

Single cells were imaged, by using the same settings for treated and untreated cells, with a Nikon Swept Field Confocal microscope (Nikon Instruments Inc.) equipped with a CFI Plan Apo VC60XH objective and an Andor DU885 EM-CCD camera, which was controlled by NIS Elements 3.2. Fifty-one-plane z-stacks were acquired with voxel dimensions of 133 × 133 × 200 nm (X × Y × Z). The mitochondrial network was then described in numbers of objects, total volume and object volume using the 3D object counter available in the software Fiji (http://www.fiji.sc) (Schindelin et al., 2012). 3D rendering was obtained with the 3D Viewer plugin.

### Measurement of ATP by Luciferase Assay

The cells were plated on 12-mm-diameter glass coverslips for single-sample luminescence measurements and incubated for 24 h. Then they were infected with a VR1012-based construct as previously described (Porcelli et al., 2001) and treated with PGZ or DMSO for further 72 h. Basal ATP content was calculated according to luminescent values of the plateau generated after luciferin addition. Efficiency of luciferase transduction in treated cells and untreated trisomic cells was determined by immunoblot assay as previously described (Izzo et al., 2014).

### Measurement of ROS on Single-Cell

Cells were seeded on glass coverslips and incubated with PGZ or DMSO for 72 h and then incubated with 2′,7′-​dichlorodihydrofluorescein diacetate (DCFH-DA, 17.5 µM) as previously described (Petrozziello et al., 2017). For DCF fluorescence analysis, each coverslip was placed into a perfusion chamber (Medical System, Co. Greenvale, NY, USA) mounted onto a Zeiss Axiovert 200 microscope (Carl Zeiss, Germany) equipped with MicroMax 512BFT cooled CCD camera (Princeton Instruments, Trenton, NJ, USA). Each coverslip was exposed at 485-nm excitation for 10 s and the emitted light was passed through a 530-nm barrier filter.

### Mitochondria Bioenergetics Measurements

Real-time measurements of OCR were made using an XF^e^-96 Extracellular Flux Analyzer (Seahorse Bioscience, Billerica, MA, USA). Cells were plated in XF^e^-96 plates (Seahorse Bioscience) at the concentration of 25,000 cells/well. Cells were counted before and after the experiments. OCR was measured in XF^e^ media (non-buffered Dulbecco’s modified eagle medium (DMEM) medium containing 10 mM glucose, 2 mM L-glutamine and 1 mM sodium pyruvate) under basal conditions and in response to 5 μM oligomycin, 1.5 μM of carbonyl cyanide-4-(trifluoromethoxy) phenylhydrazone (FCCP) and 1 μM of Antimycin-A and Rotenone (all from Sigma-Aldrich) as previously described (Izzo et al., 2017b). Each sample was plated at least in triplicate.

### Statistical Procedures

Unless otherwise indicated, all assays were performed independently and in triplicate. Statistical analysis was performed using GraphPad Prism software vers.5.0 (GraphPad Software, La Jolla California USA, http://www.graphpad.com). Student’s t test was applied to evaluate the statistical significance of differences measured throughout the data sets presented. The threshold for statistical significance (p-value) was set at 0.05.

## Results

In order to understand the impact of PGZ on mitochondrial function we exposed DS-HFF cells to different concentrations of the drug for 72 h. According to PGZ half maximal effective concentration [EC_50_ = ∼500–600 nM for both human and murine PPAR-γ activation (Sakamoto et al., 2000; Willson et al., 2000)] and previous reports in which the effects of others TZDs were analyzed (Hofer et al., 2014), we tested four increasing PGZ concentrations between 0.25 and 2 μM in DS-HFFs. Higher doses were not tested because they exert inhibitory effects on the mitochondrial respiratory chain (Garcia-Ruiz et al., 2013).

### PGZ is Not Toxic in Trisomic Cells and Induces PGC-1α Expression

To evaluate the potential toxicity of PGZ in trisomic cells, we evaluated the cell growth by MTT assay, the live/dead cell viability using the Trypan Blue dye exclusion test and the caspase activity by Western blot after 72 h of treatment at the intermediate and the highest concentrations (0.5 and 2 μM).

**Figure 1:**
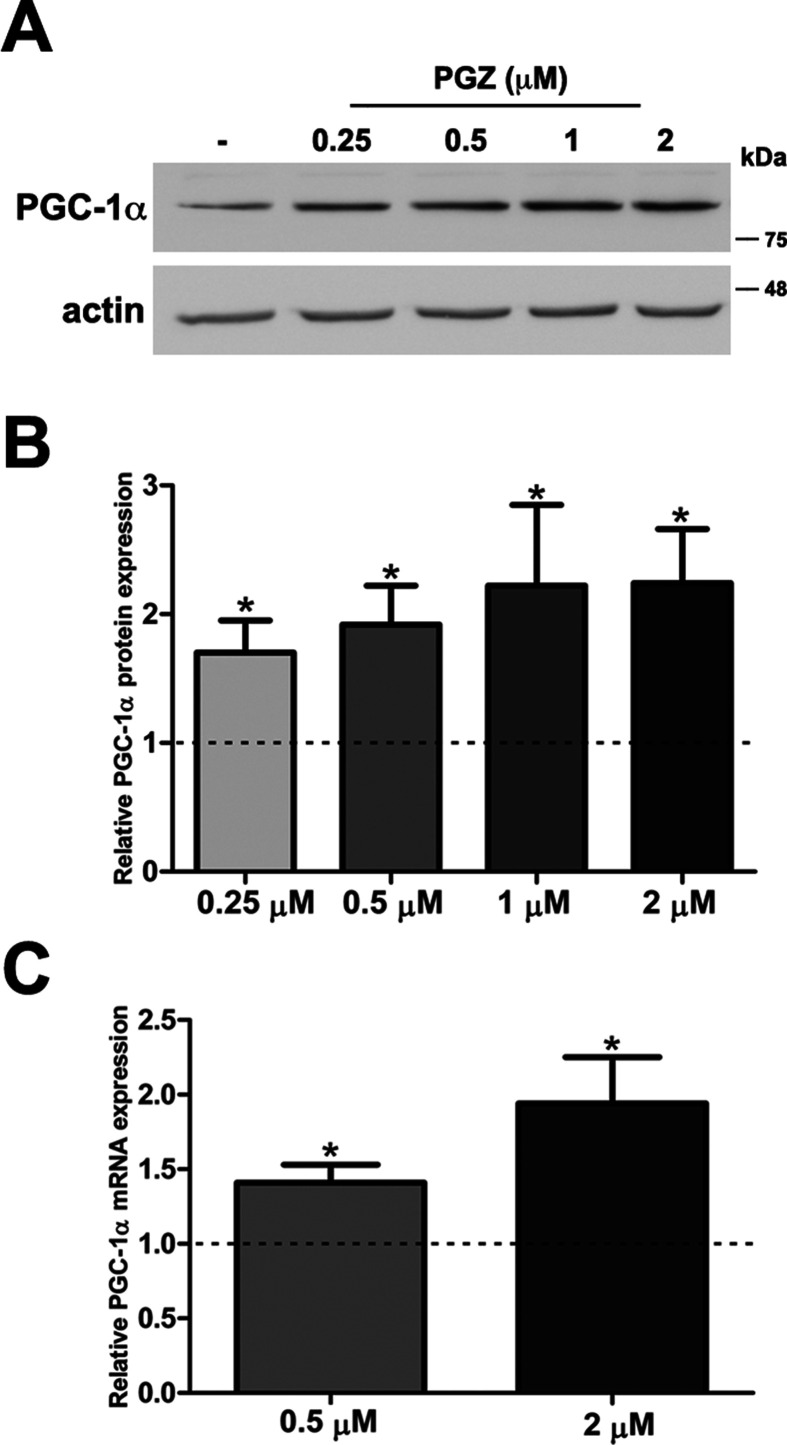
Pioglitazone (PGZ) induces PGC-1α expression in DS-HFFs. **(A)** Representative immunoblotting of PGC-1α in trisomic cells after 72 h of exposure to PGZ compared with untreated trisomic cells (-). Actin was used as a loading control. **(B)** Densitometric analysis of three different experiments is shown. Results are expressed as relative mean values ± SEM from three trisomic samples treated with PGZ, compared with untreated trisomic cells (set to 1, dashed line). **(C)** Relative *PGC-1α* mRNA expression by qRT-PCR upon normalization to a reference gene (*GAPDH*). For each trisomic sample, values represent the average measurements from two qRT-PCR experiments. Results are expressed as relative mean values ± SEM from four trisomic samples treated with PGZ, compared with untreated DS cells (set equal to 1, dashed line). *p ≤ 0.05 for comparison with untreated cells.

MTT assay is a sensitive indicator of the cellular metabolic activity being dependent on mitochondrial respiration (Mosmann, 1983). No significant variation in cell viability was found after exposure to the drug at both concentrations in comparison with trisomic cells treated with solvent only (**Figure S1A**). To exactly determine the number of viable cells after treatment, we used Trypan Blue dye. We found that the percentage of dead cells for each condition was around 2% in PGZ treated trisomic cells and controls, indicating that PGZ does not induce cellular death (**Figure S1B**). In addition, we evaluated in these cells the activity of Caspase 3, one of the most relevant biochemical markers of cell death. We did not find cleavage of caspase 3 in any of the conditions (**Figure S1C**). PGZ significantly increased the PGC-1α protein levels starting from 0.25 μM (**Figure 1A, B**). Consistently, *PGC-1α* mRNA levels were significantly increased in trisomic cells after PGZ treatment (**Figure 1C**).

### PGZ Affects the Mitochondrial Network Architecture by Modulating the Fission/Fusion Machinery

**Figure 2:**
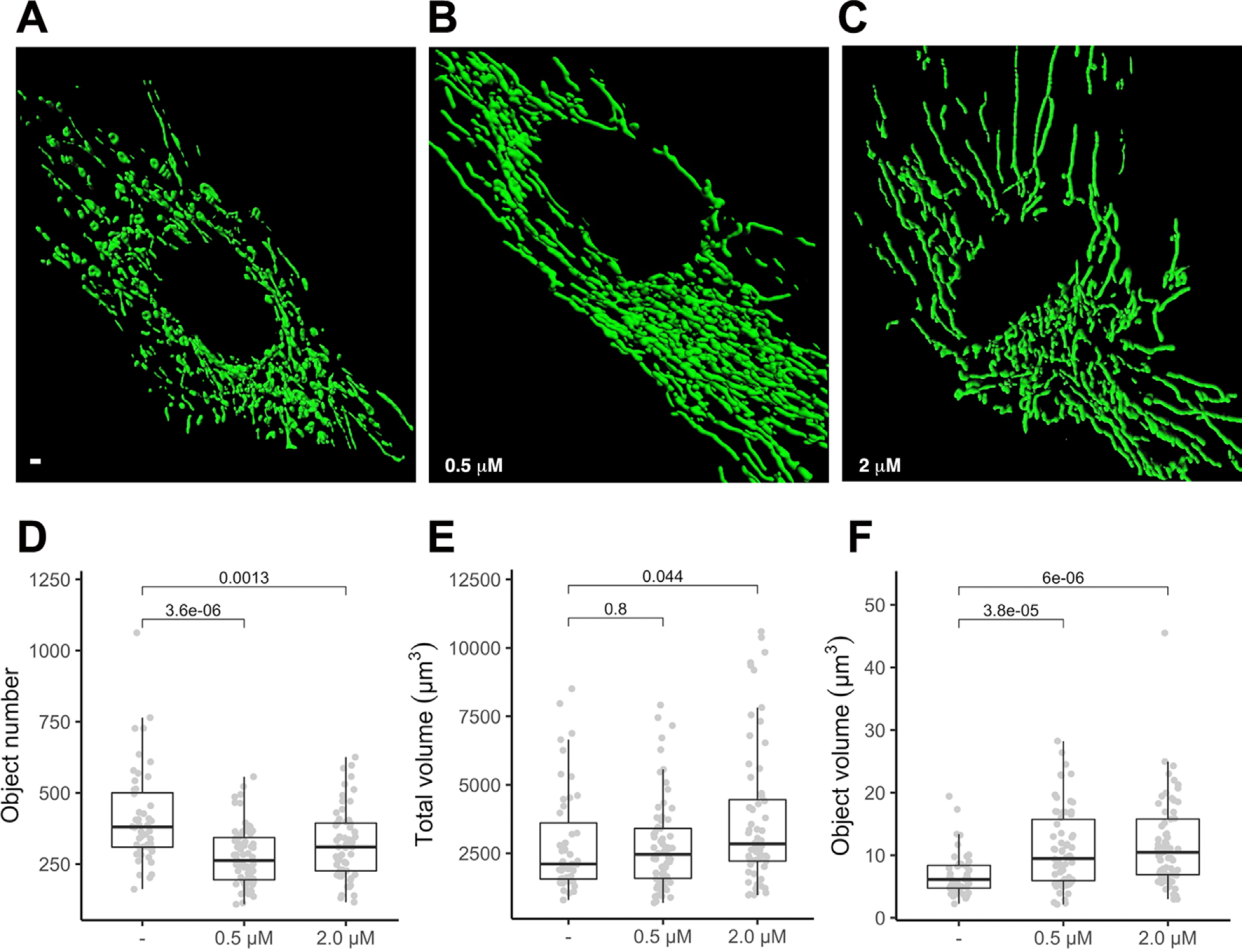
Pioglitazone restores mitochondrial network organization in DS-HFFs. Representative images of the mitochondrial network in **(A)** untreated (-) and trisomic cells treated with **(B)** 0.5 µM and **(C)** 2 µM PGZ. 3D reconstruction of Z planes acquired by using the same settings with voxel dimensions of 133 × 133 × 200 nm (X × Y × Z). Box and whisker plots represent the number of mitochondria **(D)**, the mitochondrial total volume **(E)** and the individual mitochondrial volume **(F)** measured in PGZ-treated trisomic cells and untreated cells (-).The plots show measurements from fifty randomly selected cells from four samples analyzed in each experimental condition. P-values of comparison between untreated and treated cells are indicated on plots.

To gauge if PGZ affects mitochondrial morphology in trisomic cells, we expressed a mitochondria-targeted GFP in DS-HFFs and measured mitochondrial number and mitochondrial volume in PGZ-treated versus untreated cells. At confocal microscopy analysis PGZ-treated cells exhibited a less fragmented mitochondrial network with elongated and branched mitochondria (**Figure 2B, C**) when compared with untreated trisomic cells (**Figure 2A** **)**, thus resembling to those observed in euploid cells (Izzo et al., 2017b). In detail, treated trisomic cells showed on average a lower number of mitochondria (**Figure 2D**) and an increase of individual mitochondrial volume (**Figure 2F**) when compared with untreated trisomic cells. Total volume was significantly increased only at 2 μM PGZ concentration (**Figure 2E**). Overall mitochondrial network was restored by PGZ administration in a dose-dependent manner.

**Figure 3:**
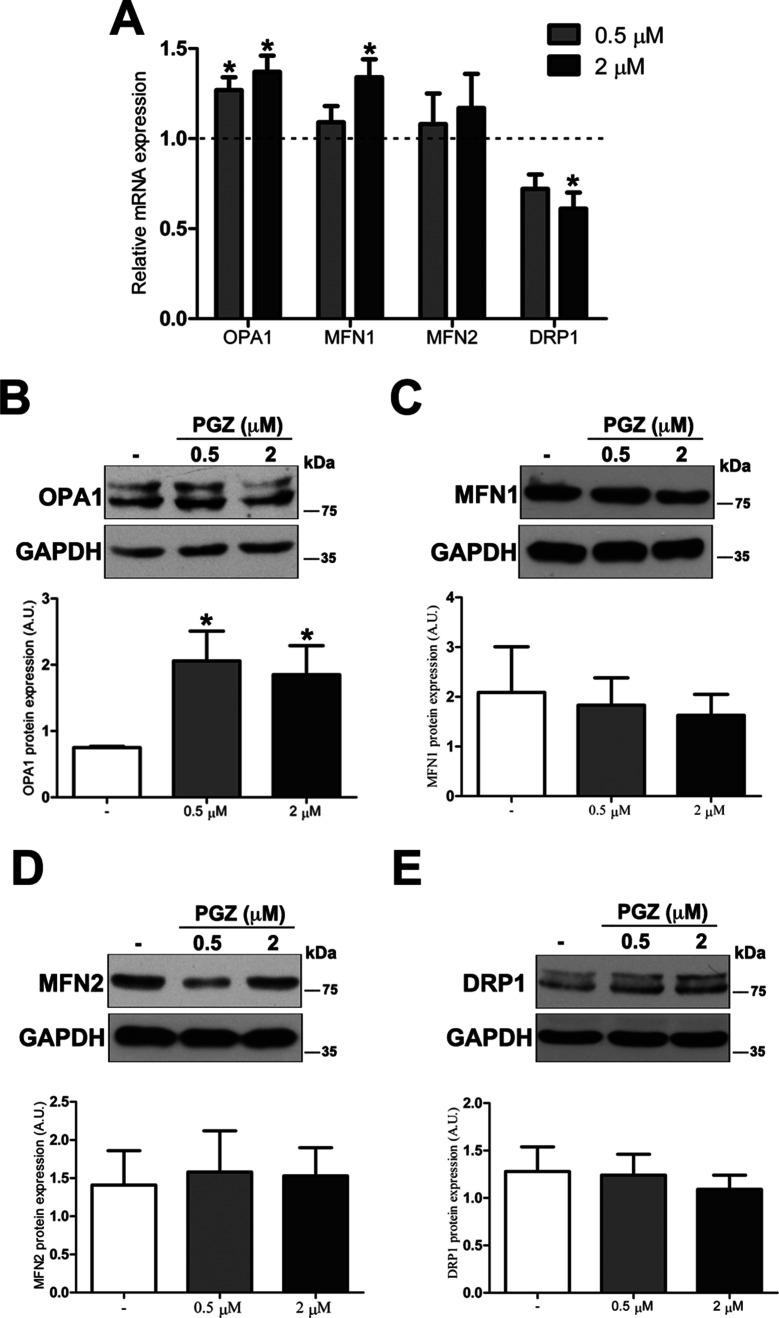
Pioglitazone modulates the expression of mitochondrial fission-fusion genes in DS-HFFs. **(A)** Relative *OPA1, MFN1*, *MFN2*, and *DRP1* mRNA expression after 72 h of treatment with PGZ (0.5 and 2 μM) measured by qRT-PCR upon normalization to a reference gene (*GAPDH*). For each trisomic sample, values represent the average determination for 2 qRT-PCR experiments. Results are expressed as relative mean values ± SEM of cell cultures from four trisomic samples treated with PGZ, compared with untreated DS cells (set equal to 1 = dashed line). Representative immunoblotting of **(B)** OPA1, **(C)** MFN1, **(D)** MFN2, and **(E)** DRP1 after 72 h of treatment with PGZ and densitometric analysis of four different experiments are shown. GAPDH was measured as a loading control. Results are expressed as mean values ± SEM from trisomic samples treated with PGZ and from untreated cells. *p ≤ 0.05 for comparison with untreated cells.

To further investigate molecular bases of the increased connectivity mediated by PGZ, the expression level of genes responsible for mitochondrial fusion (*OPA1, MFN1*, and *MFN2*) and fission (*DRP1*) was assessed. The treatment with PGZ significantly upregulated *OPA1* expression at both 0.5 and 2 μM concentrations and *MFN1* expression at 2 μM concentration (**Figure 3A**). No variation of *MFN2* gene expression was found, while the mRNA of *DRP1* was significantly decreased.

At the protein level, only OPA1 expression was significantly increased in treated cells (**Figure 3B**). Neither mitofusins nor DRP1 showed significant variation at the protein level (**Figure 3C–E**).

### Bioenergetics of Trisomic Cells Is Improved by PGZ Treatment

**Figure 4:**
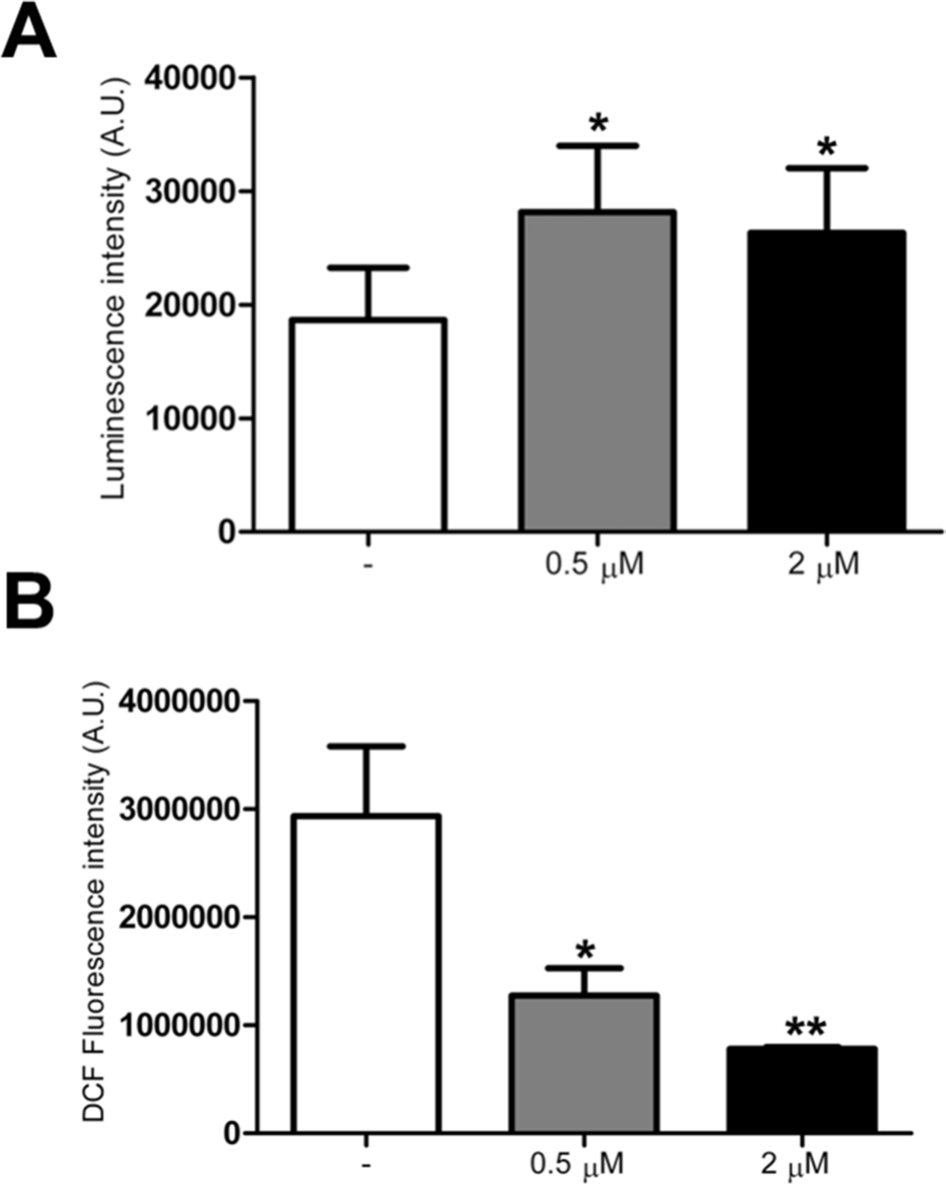
Pioglitazone improves bioenergetics and reduces ROS production in DS-HFFs. **(A)** Arbitrary units of luciferase intensity indicating basal ATP content intrisomic cells, untreated (-) and treated with 0.5 or 2 μM PGZ for 72 h. **(B)** Arbitrary units of DCF fluorescence incubated with DCFH-DA probe in trisomic cells, untreated (-) and treated with 0.5 or 2 μM PGZ for 72 h. The bars show mean values ± SEM from four trisomic samples. *p ≤ 0.05; **p ≤ 0.01 for comparison with untreated cells.

Since ATP content was previously found to be decreased in trisomic cells (Izzo et al., 2017b), using a mitochondria targeted luciferase we measured the ATP content in treated and untreated trisomic cells. A 60% increase in basal ATP content, calculated by the luminescence values of the plateau generated after the addition of luciferin, was detected in trisomic cells treated at 0.5 and 2 μM PGZ doses (**Figure 4A**). The luciferase transduction, determined by immunoblot assay, was comparable between treated and untreated trisomic cells (data not shown).

As trisomic cells display an increased ROS production with a larger redox imbalance when compared with euploid cells (Piccoli et al., 2013), we investigated the effects of PGZ on intracellular ROS in trisomic cells using the redox-sensitive probe DCFH-DA. Since ROS measurement by DCFH is affected by the duration and intensity of light excitation, the time of exposure at 485 nm was fixed at 10 s for all the samples.

DCF associated fluorescence was significantly decreased in trisomic cells after treatment with 0.5 and 2 μM PGZ for 72 h in a dose-dependent fashion (**Figure 4B**).

**Figure 5:**
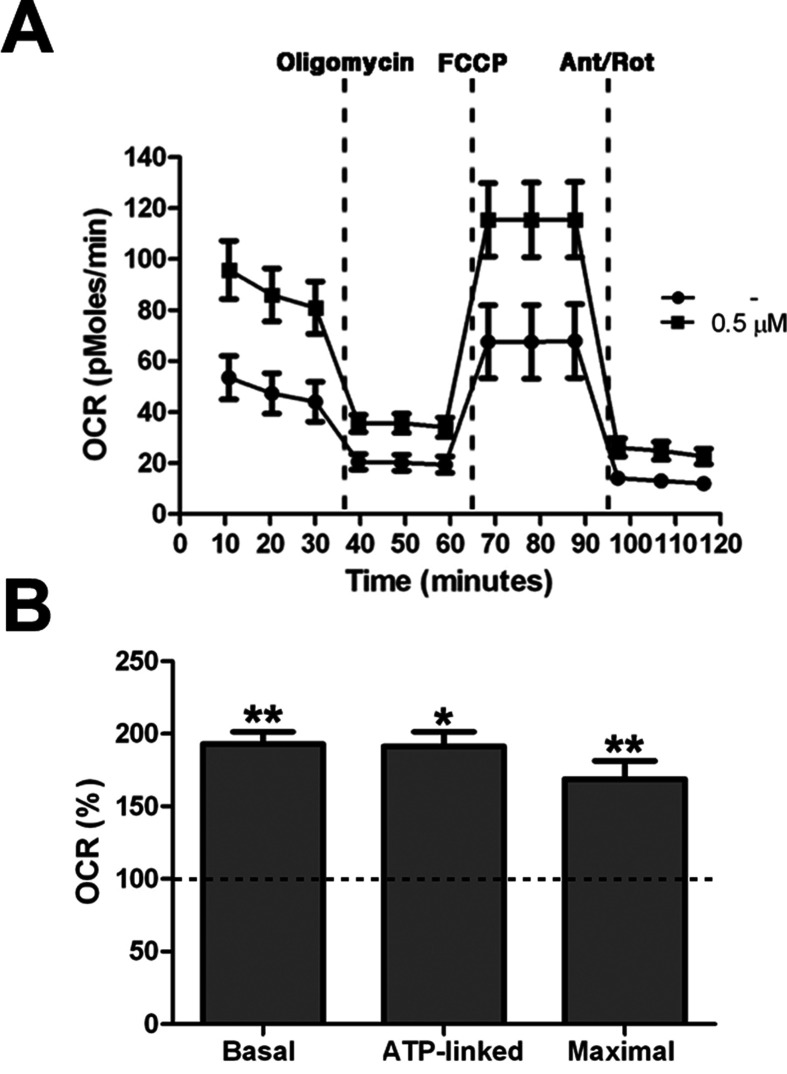
Pioglitazone improves mitochondrial respiratory function. **(A)** OCR measurement in trisomic cells after 72 h of exposure to PGZ (0.5 μM) compared with untreated trisomic cells (-). Merged curves of mean values of OCR in cell cultures from four trisomic samples obtained in basal condition and after consecutive addition of oligomycin, FCCP and Antimycin-A/Rotenon. Each experiment was carried out at least in triplicate. **(B)** Indices of mitochondrial respiratory function (basal OCR, ATP-linked and maximal) were measured. Results are expressed as relative mean values ± SEM from four trisomic samples treated with PGZ, compared with untreated trisomic cells (set equal to 100 = dashed line). *p ≤ 0.05; **p ≤ 0.01 for comparison with untreated cells.

All together these results demonstrated an increase of mitochondrial bioenergetics in treated cells, which is significant already at low concentration of the drug. As confirmation of these data we directly measured Basal OCR, ATP-linked respiration, and maximal respiration by XF^e^-96 Extracellular Flux Analyzer (Seahorse Bioscience). Cells exposed to 0.5 μM PGZ showed significant increase of all the parameters when compared with untreated cells (**Figure 5A, B**).

## Discussion

Although the phenotype of DS is complex, intellectual disability and an early development of AD neuropathology occur in almost all the individuals affected by DS (Gardiner, 2015). Increasing evidence indicates that mitochondrial dysfunction, observed in DS, contributes to generating these phenotypic traits, as mitochondrial dynamics and metabolism play an important role in both fetal and adult neurogenesis (Beckervordersandforth et al., 2017; Khacho and Slack, 2018). Accordingly, DS is associated with impairment in several structural/cognitive domains. Fetuses with DS exhibit a reduced number of neurons and a relative increase in the number of astrocytes. The brains of fetuses with DS show weight (Guihard-Costa et al., 2006) and volume reduction in various hippocampal structures (Guidi et al., 2008) and in the cerebellum (Guidi et al., 2011). Developmental defects in the dentate gyrus, altered dendritic spine morphology and reduced spine and synaptic density have also been observed (Guidi et al., 2018). These last features have been associated with the astrocyte-secreted thrombospondin 1 (Torres et al., 2018). Recent studies also demonstrated an important role of *PGC-1α* and of its targets in the formation and maintenance of hippocampal dendritic spines and synapses (Cheng et al., 2012). Impaired PGC-1α activity is emerging as a common underlying cause of mitochondrial dysfunction in neurodegenerative diseases such as AD, PD and Huntington disease (Correira, 2018). *Pgc-1α* (-/-) mice showed a decreased mitochondrial number and respiratory capacity and vacuolar lesions in the central nervous system (Leone et al., 2005). All these evidences support the hypothesis that targeting mitochondrial dysfunction in DS could help to counteract cognitive impairment and that pharmacologically induced transcriptional activation of the *PGC-1α* pathway is expected to exert neuroprotective effects.

In a previous study we successfully tested the biguanide metformin as PGC-1α activator and mitochondrial “benefactor” in a cell model of DS. Metformin enhanced OCR and ATP production promoting overall mitochondrial activity and biogenesis. The treatment also reversed the fragmentation of mitochondria observed in DS and increased the expression of genes of the fission/fusion machinery, namely *OPA1* and *MFN2* and caused a remodelling of mitochondrial cristae, thus correcting the anomalies observed by electron microscopy in trisomic cells (Izzo et al., 2017b). In the clinical practice, metformin is a well-tolerated drug but some side effects may occur (Krentz et al., 1994; Belcher et al., 2005), suggesting that alternative or synergistic possibilities may help to reduce even these mild side effects. For this reason, we have tested in a cell model of DS the effects of a thiazolinedione, namely PGZ, a PPAR-γ agonist, which stimulates *PGC-1α* expression. PGZ plays a neuroprotective role in dopaminergic neurons in both *in vitro* and *in vivo* PD models (Wang et al., 2017), which was associated with PGC-1α induction and the regulation of proteins involved in mitochondria function (Wang et al., 2017). We intentionally used low doses of the drugs because both metformin and PGZ are known to inhibit respiratory complex I when used at high concentrations (Brunmair et al., 2004; Ghosh et al., 2007; Garcia-Ruiz et al., 2013). Therefore, we tested the effects of doses that are possibly below those inhibiting the respiratory chain, as we previously did for metformin. These doses demonstrated to be non-toxic at all, as they did not affect cell viability in DS-HFFs exposed for 72 h to the drug and to enhance both expression and activity of PGC-1α in DS-HFFs. The data are compatible with the concept that the activation of PPAR-γ by PGZ induces PGC-1α expression (Villena, 2015).

Mitochondria are dynamic organelles, which readily adapt to changes in cellular energy demands through network remodeling by continuous fusion and fission processes (Benard et al., 2007; Mishra and Chan, 2016). Mitochondrial fusion results in the formation of an interconnected mitochondrial network that allows the mixing and redistribution of proteins and mtDNA (Nunnari et al., 1997). In contrast, mitochondrial fission leads to mitochondria with a fragmented morphology, which facilitates the segregation of damaged mitochondria (Berman et al., 2008; Lee and Yoon, 2016). Regulating the equilibrium between mitochondrial fusion and fission is essential to maintain mitochondrial integrity and function through the balance between mitochondrial biogenesis and degradation. These processes are intricately linked to changes in network morphology and spatio-temporal positioning of mitochondria and to their function (Hodneland Nilsson et al., 2015). They are essential for neuronal processes such as synaptogenesis, Ca^2+^ buffering, axonal transport, and bioenergetics (Oettinghaus et al., 2016).

We previously demonstrated that DS-HFFs display a mitochondrial network more fragmented with a higher number of short and globular mitochondria and a smaller average mitochondrial volume when compared with euploid controls (Izzo et al., 2017b). Mitochondrial morphology is crucial for cellular physiology, as changes in mitochondrial shape have been linked to neurodegeneration, lifespan and cell death and highlights the importance of targeting therapeutically mitochondrial dynamics (Campello and Scorrano, 2010). In the present study, trisomic cells treated by PGZ showed a network promotion and a near complete rescue of mitochondrial morphology. Analysis of genes involved in fission and fusion processes demonstrated that fusion inducing genes, downregulated in DS-HFFs, were increased by PGZ administration, while the fission inducing *DRP1* gene, over-expressed in trisomic cells (Valenti et al., 2017), was downregulated by PGZ treatment. Protein expression of this gene was too variable and yielded no statistical significance thus indicating that post-transcriptional regulating events may have occurred in the time frame that we have tested. PGZ increased fusion protein Opa1 and Mfn2 expressions and decreased fission protein Drp1 expression also in the remnant kidney of nephrectomized rats (Sun et al., 2017). A relationship between *PGC-1α* expression and mitochondrial fusion genes has been previously reported (Soriano et al., 2006; Rai et al., 2014) and silencing of *PGC-1α* in smooth muscle cells is known to increase mitochondrial fragmentation (Ryan et al., 2013). These results, together with the rescue of mitochondrial morphology observed in treated cells, can be considered a proof that mitochondrial dynamics, altered in trisomic cells, is restored by the treatment.

The functional purpose of mitochondrial biogenesis is to maintain mitochondrial quality and secure sufficient ATP production. We previously demonstrated in trisomic cells decreased ATP production, reduced oxygen consumption, increased ROS production and increased levels of intra-mitochondrial calcium (Piccoli et al., 2013). PGZ treatment induced a recovery of mitochondrial respiration in DS-HFFs. In agreement with this finding, the direct measure of the basal ATP content demonstrated that PGZ treatment was associated to a significant increase of ATP in treated DS-HFFs. We speculate that this increase may be also affected by the increase of the adenine nucleotide translocators *ANT1/SLC25A4* and *ANT2/SLC25A5*, previously found upregulated in response to *PGC-1α* induction (Izzo et al., 2014). An inverse relationship between *ANT1/SLC25A4* and the Hsa21 miRNA *let-7c*, which is upregulated in trisomic cells, has also been demonstrated (Izzo et al., 2017a). These data, together with a significant decrease of ROS production, provide strong evidence of a global improvement in energy metabolism and proof that PGZ is able to improve mitochondrial activity even at concentrations as low as 0.5 µM. This is important because higher doses deal with the risk of starting to inhibit mitochondrial complex I, as discussed above.

The complex neurological function is the result of many molecular, cellular and environmental events that are either initiated or completed before birth and must be coordinated at precisely the right time (Bartesaghi et al., 2015). This suggests that the overall impact of the neurological deficits in DS may be lessened if the initial pathologic changes in the brain are prevented from occurring. Permanent brain alterations of DS originate during fetal life in which the first window of opportunity for cognitive improvement occurs. Early prenatal diagnosis offers a temporal window to have a positive impact on brain development and to improve postnatal cognitive outcome in affected individuals. Only a few approaches (Epigallocatechine gallate, NAP/SAL, fluoxetine, and apigenin) have been used to treat mice *in utero*; some of these showed therapeutic effects that persisted to adulthood (Nakano-Kobayashi et al., 2017). Unfortunately, there is not enough information about safety of PGZ administration during pregnancy, even though no fetal malformation has been reported in the few monitored cases. Poor or discordant outcomes, controversial results of preclinical evaluations in mice and different degrees of rescue of neurogenesis obtained with previously tested antioxidants and nutraceutics (Izzo et al., 2018) suggest that combination of drugs may be both necessary and advantageous. Drug combinations offer potentially higher efficacy with lower individual drug dosage and have been considered to be beneficial in the treatment and management of other chronic medical conditions (Taylor, 2004; Muayqil and Camicioli, 2012). Different combinations of PGZ and metformin are already available in the clinical practice to improve glycemic control in patients with type 2 diabetes, with side effects similar to those that occur in mono-therapy. However, we suppose that the doses we need to govern mitochondrial dysfunction in DS could be lower than those commonly used for diabetes therapy. Further experiments will be needed to assess the smallest effective doses in a combinatory drug therapy.

## Data Availability Statement

All datasets generated for this study are included in the manuscript and the supplementary files.

## Ethics Statement

Human samples utilized in this study were obtained from the “Telethon Bank of Fetal Biological Samples,” at the University of Naples Federico II, whose activities were approved by the local Institutional Ethics Committee in November 2001.

## Author Contributions

NM, AI, SP, and AC designed the research and wrote the manuscript. NM, MN, LZ, DF, TM, RA, and TP contributed to the methodology. FB and VS conducted the statistical analysis. AS, GC, RC, and RG contributed to the data curation. GM, PP, and LN contributed to the funding acquisition and project administration. AI, AC, and LN supervised the research and revised the manuscript.

## Funding

LN was supported by grants POR Campania FSE 2007–2013 Project CREME from Campania Region, POR Campania FSE 2014–2020 from Campania Region. GM was supported by grants from European Research Council Grant “menTORingTregs” n. 310496, Fondazione Italiana Sclerosi Multipla (FISM) n. 2016/R/18 and Telethon n. GGP17086.

## Conflict of Interest Statement

The authors declare that the research was conducted in the absence of any commercial or financial relationships that could be construed as a potential conflict of interest.
