# Corrigendum: Pioglitazone Improves Mitochondrial Organization and Bioenergetics in Down Syndrome Cells

**DOI:** 10.3389/fgene.2020.00728

**Published:** 2020-07-27

**Authors:** Nunzia Mollo, Maria Nitti, Lucrezia Zerillo, Deriggio Faicchia, Teresa Micillo, Rossella Accarino, Agnese Secondo, Tiziana Petrozziello, Gaetano Calì, Rita Cicatiello, Ferdinando Bonfiglio, Viviana Sarnataro, Rita Genesio, Antonella Izzo, Paolo Pinton, Giuseppe Matarese, Simona Paladino, Anna Conti, Lucio Nitsch

**Affiliations:** ^1^Department of Molecular Medicine and Medical Biotechnology, University of Naples Federico II, Naples, Italy; ^2^Department of Morphology, Surgery and Experimental Medicine, University of Ferrara, Ferrara, Italy; ^3^Institute of Experimental Endocrinology and Oncology, National Research Council, Naples, Italy; ^4^Department of Biology, University of Naples Federico II, Naples, Italy; ^5^Department of Neuroscience, Reproductive and Odontostomatological Sciences, University of Naples Federico II, Naples, Italy; ^6^Department of Biomedicine, University of Basel, Basel, Switzerland

**Keywords:** Down syndrome/therapy, pioglitazone, energy metabolism, oxidative stress, mitochondrial dysfunction, mitochondrial dynamics

In the original article, there was an error in the Funding statement. The correction has been made to the Funding section by removing a funder “Lejeune Project #1698”:

LN was supported by grants POR Campania FSE 2007–2013 Project CREME from Campania Region, POR Campania FSE 2014–2020 from Campania Region. GM was supported by grants from European Research Council Grant “menTORingTregs” n. 310496, Fondazione Italiana Sclerosi Multipla (FISM) n. 2016/R/18 and Telethon n. GGP17086.

The authors apologize for this error and state that this does not change the scientific conclusions of the article in any way. The original article has been updated.

