# Supplementary material for: Pioglitazone Improves Mitochondrial Organization and Bioenergetics in Down Syndrome Cells
Source: Front Genet. 2019 Jun 28;10:606. doi: 10.3389/fgene.2019.00606 (PMC6609571; doi:10.3389/fgene.2019.00606)
Supplement: Supplementary file 1 [file Table_1.docx]

Supplementary Material

**Supplementary Materials**

**Drug Toxicity**

To quantify the potential toxicity of PGZ, mitochondrial activity was evaluated by the MTT (3[4,5-dimethylthiazol-2-yl]-2,5-diphenyl-tetrazolium bromide) that is metabolized by viable mitochondria to a colored product detected at a wavelength of 540 nm. Briefly, after 72 hrs drug treatment, 1 mL MTT were added to each well for 1 h at 37° C as previously described (Petrozziello, 2017). Data were expressed as % viability rate in comparison with untreated trisomic cells.

**Trypan blue assay**

The cells were plated in 6-well plates (30000 cells/well) and treated for 72 hrs with solvent only or PGZ. After treatment cells were stained using 0,4% Trypan blue dye (Biorad) to determine dead cells. Cell count was performed manually with a hemocytometer in two trisomic samples in duplicate. Data are expressed as percentage of live and dead cells on the total.
