# Supplementary figures and images for: Pioglitazone Improves Mitochondrial Organization and Bioenergetics in Down Syndrome Cells
Source: Front Genet. 2019 Jun 28;10:606. doi: 10.3389/fgene.2019.00606 (PMC6609571; doi:10.3389/fgene.2019.00606)

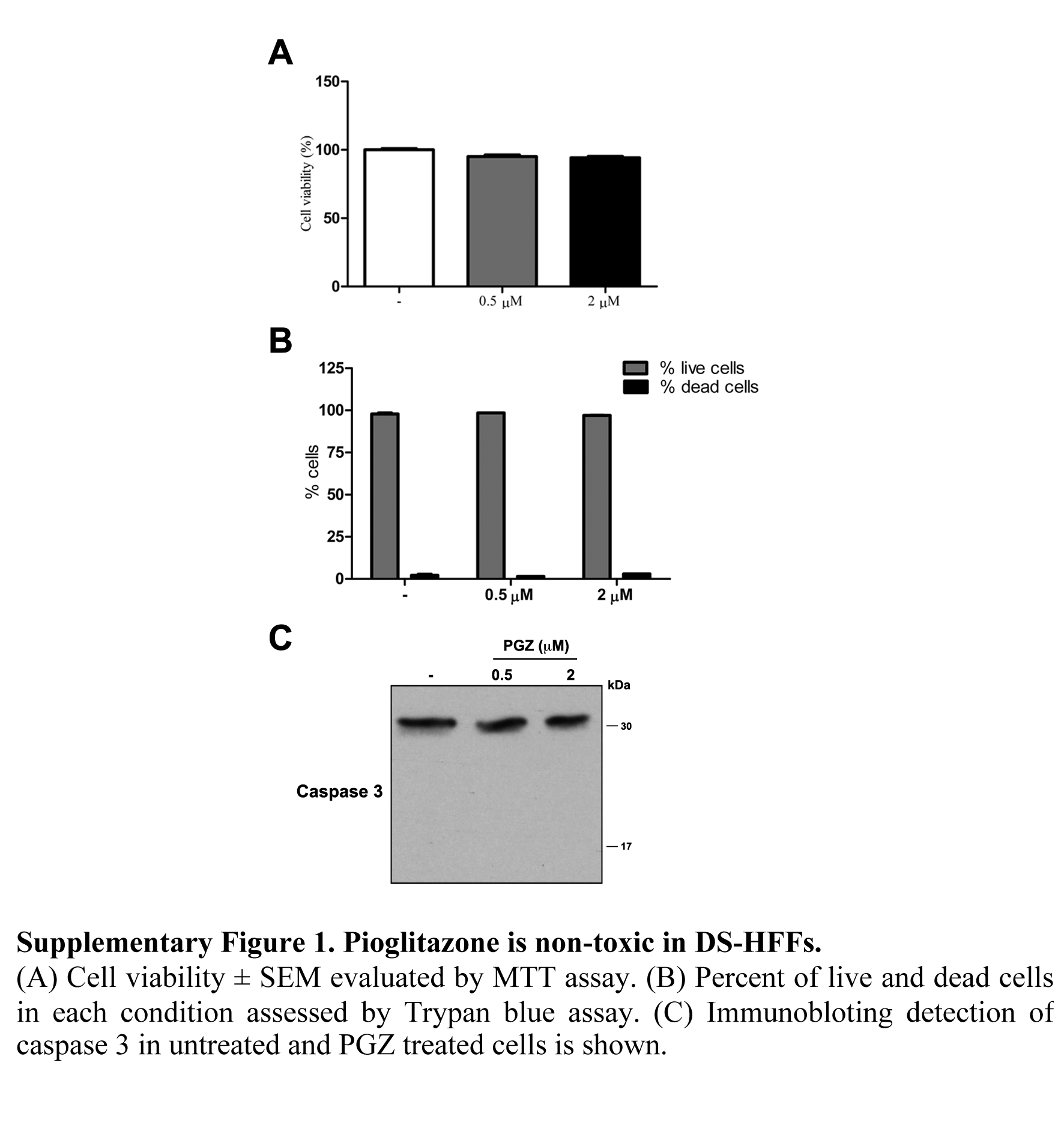

Supplement: Supplementary file 2 [file Image_1.tif]
